# Lessons Learned From Blue Zones, Lifestyle Medicine Pillars and Beyond: An Update on the Contributions of Behavior and Genetics to Wellbeing and Longevity

**DOI:** 10.1177/15598276221118494

**Published:** 2022-08-20

**Authors:** Magdalini Kreouzi, Nikolaos Theodorakis, Constantina Constantinou

**Affiliations:** Department of Basic and Clinical Sciences, 486462 University of Nicosia Medical School, Nicosia, Cyprus (MK, CC); Department of Internal Medicine, 218772 Limassol General Hospital, Limassol, Cyprus (NT)

**Keywords:** Blue Zones, lifestyle medicine, wellbeing, genetics, longevity

## Abstract

Blue Zones are regions of the world that have a higher number of individuals who live longer than the expected average. The current paper revisits principles previously identified to be common in Blue Zones and to be contributing to longevity (*move naturally, eat wisely, improve resilience to stress, get adequate sleep, keep strong family ties, stimulate strong community support, respect for the planet* and *having a purpose in life’*), compares these to the 6 pillars of Lifestyle Medicine (*healthy eating*, *exercising*, *avoidance of smoking and other risky substances*, *stress management*, *restorative sleep, and forming and maintaining relationships)* and reviews new studies investigating the association between behavioral factors and longevity. In addition to the role of behavior, the review also discusses the important role of genetics and emphasizes the importance of conducting further research to understand how behavioral and genetic factors may affect molecular pathways with consequent effects on wellbeing and longevity.

“In addition to the role of behavior, genetics seems to play an important role in affecting longevity and some of the genes involved include FOXO3A, ApoE2 and HLA.”

## Introduction

Blue Zones (BZ) have been defined as regions of the world that have a higher number of individuals who live longer than the expected average. The first reported BZ described was Sardinia (Barbagia Region) in 2004 and subsequently 3 additional regions were reported including Okinawa (Japan), Nicoya (Costa Rica) and Ikaria (Greece).^1,2^

Based on the definition given by Poulain, a longevity BZ is defined as: “an area where the population is characterized by a significantly higher level of longevity compared to neighboring regions and the exceptional longevity of people in this population must be fully validated”.^3^ For the validation of population longevity several indexes are used which include Centenarian Prevalence, Centenarian Rate and Extreme Longevity Index, which serve as objective measurement of longevity.^3^

According to the World Health Organization (WHO), the global average for life expectancy is 73 years of age (71 for males and 76 for females).^4^ The life expectancy averages in all BZ have not been established by credible sources; in these areas the proportions of people living above 90 or reaching centenarian age is significantly higher than the general population.^1^ Researchers have identified several factors that influence peoples’ longevity in the BZ regions. Poulain and Herm (2022) identified factors which are common in all BZ and could contribute to the longevity of those populations and were labeled as “*Move naturally,” “Eat wisely,” “Avoid stress,” “Get plenty of sleep,” “Keep strong family ties,” “Stimulate strong community support,” “Respect for the planet,” and “Having a purpose in life.”*^1^ However, despite subsequent studies in the BZ, up to date there has not been a specific scientific explanation regarding the exceptional advantage of survival of the inhabitants of BZ and the latter is likely the result of a combination of various behavioral and genetic factors.^2,3,5-11^

Noncommunicable diseases (NCD) are on an ascending slope internationally. 71% of global deaths are due to NCD, the majority of which being attributed to coronary artery disease, stroke, cancer and diabetes mellitus.^12^ The majority of the risk factors associated with these diseases are modifiable; it has been shown that 50% of premature deaths in developed countries such as the United States are due to modifiable lifestyle behavioral risk factors such as smoking, nutrition and exercise.^13^ Lifestyle Medicine (LM) is a rapidly developing field which studies the lifestyle-related risk factors of noncommunicable diseases and the ways of modifying them in order to improve the prolong life expectancy, decrease the burden of chronic disease and improve the quality of life of individuals. LM addresses 6 lifestyle pillars which include *healthy eating, exercising, avoidance of smoking and other risky substances, stress management, restorative sleep, and forming and maintaining relationships.*^14^

The aim of this review is to revisit lessons learned from Blue Zones, compare these to the LM pillars and discuss emerging evidence on the association between genetics and metabolic mechanisms with wellbeing and longevity.

## Comparing the Lessons Learned From the Blue Zones to the Pillars of Lifestyle Medicine

In the current section we compare the 7 principles for living longer in the BZ to the 6 Pillars of LM, while reviewing recent studies conducted in related fields.^1,14^

## Healthy Eating

### Plant Based Diets and Caloric Restriction

Diets in the BZ are based on consumption of healthy real food which is mostly plant based and this is how the *“Eat wisely”* factor by Poulain and Herm was derived.^1^ Okinawans use a 2500-year-old Confucian mantra called Hara bachi bu which establishes the ‘*80% rule’*. The above reminds people before their meal that they should stop eating when their stomach is 80% full.^1^ Therefore, Okinawans consume a diet characterized by a moderate caloric restriction (10-15% restriction in calories), while they also consume caloric restriction mimics, that is, foods that have molecular effects similar to caloric restriction, such as sweet potatoes, marine-based carotenoid-rich foods, and turmeric. Caloric restriction leads to a decrease in the ATP:AMP ratio activating the AMP-activated protein kinase (AMPK) which has a variety of functions including inhibition of the mechanistic target of rapamycin (mTOR) and the insulin-like growth factor-1 (IGF-1) pathways, lipolysis, increased insulin sensitivity, autophagy, anti-oxidant, anti-neoplastic. Anti-inflammatory and anti-aging effects.^15^ Even though caloric restriction is considered a longevity-promoting diet, apart from Okinawa the average caloric intake of the inhabitants of other BZ does not significantly differ from the general population’s intake.^6,15,16^

Nicoyans and Sardinians from Oligastra consume a mainly plant-based diet with plenty of cereal and legumes, complemented mostly by dairy products.^17^ As far as plant-based diet is concerned, its connection to longevity is partly due to the abundant consumption of fruits and vegetables, which are protective factors against CVD and many types of cancer, as well as the absence of red meat from the diet, which is rich in saturated fats and increases the risk of CVD and some types of cancer, particularly gastrointestinal.^17,18^ However, plant-based diets are less balanced and may lead to deficiencies (eg, vitamin B12 deficiency in strictly vegan diets, iron deficiency in premenopausal women), while caloric restriction poses a risk of malnutrition and muscle wasting.^19^

The molecular effects of plant-based dieting have been studied in a sub-population (n = 96) of the Adventist Health Study-1 (AHS-2), a large cohort (n = 96 000) study evaluating the health effects of vegetarian diets. One major category of epigenetic regulators are micro-RNAs (miRNAs), which are small molecules which regulate the transcription of a variety of genes. The study investigated the presence of several dietary-regulated miRNAs, which regulate many age-related cellular activities in the 96 participants of the study. The results showed the vegetarian diets have a major effect on the expression of miRNAs involved in the protection against many age-related diseases, such as Alzheimer’s Disease, Parkinson’s Disease, connective tissue diseases and thrombotic diseases with these associations being stronger in males.^20^

Interestingly, the Okinawan diet includes Alpinia Zerumbet, a plant with promising anti-obesity, antioxidant, and anti-aging properties. Animal studies have shown that this plant could expand the lifespan of humans by 22.6%.^21^ Ooitabi extract (*Ficus pumila* L.), another plant which Okinawans consume, could possibly contribute to their longevity via its ability to improve the lipid profile, insulin sensitivity, blood pressure and uric acid levels.^22^ Other studies have shown that the Okinawan propolis has anti-aging and anti-cancer abilities via the inhibition of p21 activated kinase 1 (PAK1), while the extract from Melia azedarach L. leaves has a high cytotoxic effect against cancer cells in vitro.^23,24^

Despite reports that inhabitants of the BZ consume a primarily plant-based diet, various other studies showed that there is a variety of dietary habits between different BZ. For example, as already stated, Okinawans and Sardinians from Oligastra supplement their diet with a considerable amount of dairy products.^17^ On the other hand, Ikarians consume foods based on the traditional Mediterranean diet, which will be analyzed in the next section of the article. A recent study by Poulain et al showed that inhabitants of a Sardinian consume increasing amounts of olive oil, fruits and pasta, as well as dairy products (ricotta, casu ajedu) and poultry meat. The later may be correlated with improved functionality in elders as a means of preserved muscle mass. While meat has been associated with increased all-cause mortality and various diseases (eg, CVD, diabetes mellitus type 2, some cancers), these associations are only true for red or processed meat. Based on WHO processed and red meats are classified as group 1 and 2A carcinogens, respectively. On the other hand, white meat (fish and poultry meat) possesses significant health benefits, including increased muscle mass, strength and functionality and improved ability to efficiently participate in aerobic exercise and resistance training, with all the respective health effects.^8^

### Mediterranean Diet

The Mediterranean diet is popular in Ikaria and has been shown to have a crucial role in the reduction of morbidity and mortality. The traditional Mediterranean diet recommends the consumption of fat originating from monounsaturated fatty acids, increased consumption of fruits, vegetables, legumes, nuts, whole grain cereals, moderate consumption of fish, poultry, and wine as well as minimal consumption of red meat, sweets and foods rich in trans fatty acids. The Ikarian studies of Panagiotakos et al in 2011 (1330 participants, mean age: 64.5) and Legrand et al in 2021 (71 participants, aged ≥90) showed a 69% and 62.7% adherence to the traditional Mediterranean diet, respectively.^9,25^

Hyperuricemia (raised uric acid) has been shown to be correlated with a significantly higher risk of CVD such as arterial hypertension, stroke, congestive heart failure and other comorbidities linked with high risk of morbidity and mortality.^26^ Chrysohoou et al^27^ studied the impact of adherence to a Mediterranean diet on serum uric acid levels in 257 men (75±7 years old) and 281 women (75±6 years old) living in Ikaria and concluded that the adherence to a Mediterranean diet was associated with decrease uric acid levels in the targeted elder population of Ikaria and was more evident in males.^27^

Another positive correlation between the Mediterranean diet and longevity is associated with the consumption of fish and its role in preventing depression. The prevalence of depression in the 21^st^ century are approximately 2-12% in men and 4-25% in women and is strongly correlated with physical, social, and mental impairment.^28^ Furthermore, depression is described as a disorder with high association with morbidity and mortality.^29^ Chrysohoou et al 2011 revealed that a population of elder residents of Ikaria (n = 673, 65-100 years old) which had a long-term consumption of fish had a much lower risk of developing depressive symptoms.^30^

#### Olive Oil

Exclusive olive oil consumption, a major component of the Mediterranean diet, is widely known for its cardioprotective effects.^31^ These effects may be attributed to antioxidant and anti-inflammatory properties of monounsaturated fatty acids, Vitamin E and phenols found in olives.^32^ The GREECS observational study concluded that the use of non-exclusive olive oil consumption was associated with a 40% increase in the risk of development of fatal or nonfatal acute coronary syndrome when compared to exclusive olive oil consumption, thereby outlining the important role of olive oil in the prevention of CVD.^31^

#### Greek Coffee

Studies conducted in Ikaria have shown that moderate consumption of Greek coffee decreases the risk of CVD. This beneficial effect may be contributed to the anti-inflammatory and antioxidant properties of coffee which includes chlorogenic acid, flavonoids, vitamin E, vitamin B3, magnesium and potassium. One important property of Greek coffee is its beneficial effects on the endothelial function of blood vessels which is directly related to reducing the risk of developing CVD. Moreover, the *National Institutes of Health-AARP Diet and Health Study* concluded that moderate coffee consumption decreases total mortality after adjusting for smoking and other possible confounders. Inhabitants of Ikaria consume Greek coffee at a rate of over 87% and this may partly contribute to their longevity.^33-35^

### Moderate Alcohol Consumption

In most BZ, drinking alcohol moderately and regularly is a tradition. Based on the study by Poulain et al, both male and female Ikarians drink statistically significantly more alcohol than inhabitants in other parts of Greece. They mainly consume red wine which is known to have possible antioxidant and cardioprotective effects when consumed in low to moderate amounts.^3^ In another study, Legrand et al, conducted a 1-year observational study with a sample population of 71 (age 90 and over) residents of Ikaria and showed that 75% of Ikarians drink 1-2 glasses of alcohol daily.^7^ Sardinians are also famous for their daily consumption of the robust, regional red wine called Cannonau, which possesses antioxidant and vasodilatory properties.^36^ However, a large ecological study reported that the average intake of red wine is equal in the Sardinian BZ and the rest of the island, where the longevity level is lower, questioning the positive impact of alcohol consumption in the extension of lifespan.^37^

Low to moderate consumption of red wine has many proven health benefits, however the recommendation of alcohol consumption should be avoided due to the addictive nature of the substance and various other side effects. For those who are consuming alcohol on a weekly basis at low to moderate amounts consistent with the recommended weekly allowances (<14 units of alcohol per week, while avoiding binge drinking) there should be a strong recommendation to substitute beer and other spirits with red wine (maximum 6 medium glasses of wine 175 mL each per week).^38^

### Common Characteristics of Healthy Eating in the BZ Compared to LM Recommendations

LM proposes that physicians should educate their patients to avoid salt, processed foods, sweets, foods rich in trans fatty acids and large amounts of red meat, and encourage the consumption of fruits, vegetables, cereals, nuts, seeds, legumes, olive oil, dairy products, fish and poultry meat. The above LM recommendations are consistent with the nutritional habits observed in the Ikarian and Sardinian BZ. These dietary characteristics offer various health benefits such as protection against obesity, diabetes mellitus type 2, metabolic syndrome, CVD and various types of cancer.^14^

## Physical Activity

One of the most important aspects of longevity in the BZ is the role of exercise in the life of centenarians. Most residents of these areas use aerobic exercise as a mean of activity for example, walking, growing a garden, household chores etc. This has derived the ‘*Move naturally’* factor described by Poulain and Herm.^1^

There are various benefits of physical activity on cardiovascular health including the improvement of prognosis in heart failure, the reduction of blood pressure, weight loss, glycemic control and the improvement of the lipid profile.^39-41^ A study conducted to a subgroup population of Ikaria (n = 327, 40-91 years old) evaluated flow mediated dilation (FMD) and total antioxidant capacity in 3 subgroups: low, moderate, and vigorous physical activity. Flow mediated dilation is an innovative marker of the endothelial function and it is based on the ability of the endothelial cells to release nitric oxide (NO) after flow restriction. The study found that the increase in physical activity was associated with a statistically significant increase in FMD (*P* = .03) and total antioxidant capacity (*P* = .01). The following findings showed that physical activity levels play a determinant role in the improvement of endothelial function and thus the conquer of oxidative stress, hence decelerating the process of atherosclerosis.^42^ The study by Panagiotakos et al showed that almost 9 out of 10 men and 7 out of 10 women reported daily physical activities (eg, gardening, walking, occupational), which is significantly higher than the average of Greek Islands and Cyprus republic (5 out of 10 men, 4 out of 10 women, as shown in MEDIS study). Furthermore, almost 6 out of 10 Ikarians over the age of 90 enrolled in the same study performed some type of physical activity, which is again much higher than the standards of MEDIS study (2 out of 10 elders in the same age group).^25^ Legrand et al showed that grip strength for all Ikarian participants was higher than average for their age which contributed to their increased muscle strength.^7^ Another Ikarian study by Legrand et al showed that 71.8% of the 71 participants aged ≥90 were physically active.^9^

Additionally, Siasos et al investigated the impact of physical activity on endothelial function in middle-aged (n = 185, 40-65 years old) and elderly individuals (n = 142, 66-91 years old) living in Ikaria. For this study endothelial function was again evaluated with the use of FMD (US guided). The results of the study demonstrated FMD was inversely associated with age with middle aged subjects exhibiting higher FMD measurements compared to elders (*P* = .003). The above highlights that middle aged physically active individuals had higher FMD measurements compared to the physically active elders. Furthermore, researchers concluded that the increase in physical activity in middle-aged individuals was associated with an increased FMD and improved endothelial function. In summary, increased physical activity may alleviate of the effects of ageing on arterial wall properties.^43^

Oikonomou et al examined the effects of physical activity on the modification of the arrhythmogenic activity of the heart in Ikarians. The QT interval on a resting electrocardiogram, representing the duration of ventricular depolarization, is usually adjusted via Bazett’s formula (QTc = QT/RR^1/2^) and poses a more accurate estimation of the risk of arrhythmias. There are various factors that can cause QTc elevation, varying from structural heart disease, myocardial infarction, drugs, and electrolyte disorders to old age. On the above-mentioned study, it was shown that middle-aged and elderly women in the vigorous PA group were 5.5 times less likely to possess a QTc above 450 ms, compared to the low PA group (*P* = .031), enlightening the preventive effect of exercise in arrhythmia development. One proposed physiologic mechanism for the above relationship is that QTc elevation may be due to an imbalance between the sympathetic and parasympathetic nervous system and physical activity may normalize and restore this problem. On the other hand, there was no statistically significant difference in the QTc duration in relation to exercise levels in males of the same study (*P* = .053).^44^ As for Okinawans, Basho-fu weaving is a part of the cultural identity of inhabitants the Ogimi village and it is a form of physical activity that most women engage to, even at old ages.^45^ Mario Pes et al showed that Sardinian nonagenarians had a great physical activity level and showed supreme physical functional indexes.^46^

### Physical Activity in the BZ Compared to LM Recommendations

The LM recommendations for exercise are consistent with the active lifestyle of individuals inhabiting the BZ. LM proposes that adults must participate in at least 150 min/week of moderate intensity aerobic activity and at least twice weekly resistance training including all muscle groups.^14^ The above recommendation is achieved only by 23% of adults in developed countries (eg, United States), while 30% of ischemic heart disease and 6-10% of the mortality of NCD is due to physical inactivity. There is therefore an imperative need for LM physicians to promote physical activity as a way of disease prevention and wellbeing.^14^ Every individual should try to meet the recommendations for aerobic and resistance-training physical activity based on the WHO 2020 guidelines.^47^

## Avoidance of Risky Substances

### Smoking

In the study by Legrand et al, almost 50% of Ikarians had been smokers with an average age of smoking cessation being 60.6 years and 7% were active smokers.^7^ In addition, the study Giovanni et al showed that 48% of Sardinian elders aged 89-101 had been smokers in the past, while only 1% were active smokers.^48^ The above findings could indicate that smoking cessation is a greatly important lifestyle change that needs to be embedded by the general population and it could greatly contribute to longevity.

### Polypharmacy

Legrand et al showed that the prevalence of polypharmacy was low in the Ikarian population of the study as only 23.9% were taking more than 3 medications daily, whereas the average of Greek elders took more than 5 medications daily.^7^ Madrigal-Leer et al showed that a low rate of polypharmacy is a common phenomenon in Nicoyans, in addition to Ikarians.^49^ The above phenomenon supports the idea that polypharmacy may be harmful for elder patients.

### Avoidance of Risky Substances in the BZ Compared to LM Recommendations

With regards to risky substances LM highlights that particularly tobacco smoking and alcohol abuse constitute a large portion of preventable deaths in the developed countries. The health effects of vaping are yet to be determined, however Campagna et al showed that tobacco smokers who switched to vaping had no health benefits. Physicians should make a substantial effort to aid in the modification of these behaviors using various strategies, such as the COACH approach (curiosity, openness, appreciation, compassion, honesty).^14^

## Stress Management and Maintenance of Good Mental Health

Stress is a worldwide issue and can lead to chronic inflammation and age-related diseases.

Among the habitants of the BZ stress may be low and this may be due to the fact that they may have found ways of managing their stress or they may have developed resilience towards stressful situations. The principle *“Avoid stress”* proposed by Poulain and Herm^1^ may be difficult to achieve since almost every human activity (eg, work, maintenance of social relationships) produces variable levels of stress as a normal physiological response. Since avoidance of stress is inevitable, managing chronic stress and improving resilience towards stressful situations may present more tangible goals.

The exact pathophysiologic mechanism linking chronic stress with increased mortality remains to be specified, however there are indications that chronic cortisol secretion can lead to arterial hypertension, central obesity, insulin resistance and subsequently metabolic syndrome and CVD. Hair cortisol has even been identified as a possible marker of chronic stress and cardiovascular health.^50^

Legrand et al, reported that depression amongst Ikarians was infrequent with an occurrence of 11.8% and that only 39.1% of the individuals had a cognitive impairment when a mini mental state examination was conducted in participants of their study.^7^ Large-scale studies have concluded that positive psychologic aspects of well-being have been associated with a decreased risk of the development of CVD and decreased advert outcomes in people suffering from CVD. However, the above association may be affected by various confounding factors, as well-being is associated with better sleep quality, healthy diet, adherence to medication, exercise and non-smoking status.^51^

Madrigal-Leer et al studied various characteristics of a sample of Nicoyan centenarians (n = 37) and concluded that 93.75% of this population were satisfied with their lifestyle and showed no signs of depression. On the other hand, the CRELES study showed that depression using the Yesvage scale ranged up to 17% in the general population of Costa Rica.^49^ Studies from Okinawa and Sardinia have concluded that the good quality of sleep, the participation in leisure activity and social interaction all contribute to the mental wellbeing of these populations, which may be directly associated with their longevity.^52,53^

### Good Mental Health in the BZ Compared to LM Recommendations

Chronic stress is a major pandemic of the developed countries in the 21^st^ century and it is usually associated with work or money. Apart from the increase in the risk of CVD, which has already been highlighted, chronic stress can trigger the first episode or exacerbations of various inflammatory or autoimmune diseases, such as atopic dermatitis, psoriasis and inflammatory bowel disease, and even impair the function of the immune system via the chronic hypercortisolemia. Furthermore, mental health is directly associated with physical health and somatic symptoms and vice versa, according to the biopsychosocial model of medicine.^14^ Since complete avoidance of stress is impossible, LM recommendations for copying with stress include a healthy diet, exercising, adequate sleep, social connections, laughter, leisure activities, sports, breathing techniques, meditation, yoga, tai chi, mindfulness-based stress reduction and the relaxation response.^14^

## Restorative Sleep

The elderly of the BZ follow the diurnal cycle of the sun by both sleeping and waking up early, contributing to the factor ‘*Get plenty of sleep’* proposed by Poulain and Herm.^1^ In their study of a subset of the Ikarian population Panagiotakos et al reported that almost all participants reported regular napping, with all of the participants over 90 years old sleeping in the noon. Furthermore it was reported that those who napped regularly had statistically significantly lower scores in the Geriatric Depression Scale (3.4 ± 3.0 vs 5.8 ± 4.2, *P* < .001).^25^ The importance of napping on longevity was highlighted by another study which included 23 681 otherwise healthy residents from Greece. This study reported that midday siesta lowered stress levels and reduced the risk of CVD mortality.^54^ Other studies have shown that poor quality of sleep has also been associated with an increased risk of developing arterial hypertension, type 2 diabetes mellitus, obesity and depression, as well as poor glycemic control in type 2 diabetics. Hypercortisolemia plays a central role in the pathophysiology of the above associations, especially in the case of type 2 diabetes mellitus, as it antagonizes the physiological actions of insulin.^55,56^ Furthermore, sleep deprivation leads to an imbalance in the hormones controlling satiety and specifically increases the levels of ghrelin and decreases the levels of leptin, leading to overeating.^14^

The importance of restorative sleep has also been confirmed by studies in the Okinawans which showed that individuals with <6h of daily sleep had increased cardiovascular and pneumonia mortality rates.^57^ Furthermore, a great majority of Okinawan elders inhabiting the suburban areas sleep adequately at both noon and night when compared to those inhabiting the urban areas.^58^

### Restorative Sleep in BZ Compared to LM Recommendations

The guidelines of the National Sleep Foundation state that adults require 7 to 9 hours of good quality sleep per night. Apart from the increase in the risk of developing CVD and metabolic syndrome, as already stated, sleep deprivation is associated with a negative impact in most body systems and even the development of cancer. However, it should be noted that sleeping over 9 hours per night should also be avoided since it might increase the risk of morbidity (eg, obesity, diabetes mellitus, cardiovascular disease) and mortality and what is mostly important is that people take adequate and restfulsleep.^59,60^ According to LM, physicians should highlight the importance of good quality sleep and adverse effects of sleep deprivation to their patients. At the same time, they should aid in the management of the roots of this problem, via stress control, pain management in chronically ill patients and treatment of concurrent mental diseases). They should also provide strategies to ensure a good quality of sleep, which may include avoidance of using light sources late at night (eg computers, mobile phones) which suppress the secretion of melatonin, introducing late afternoon exercise, ensuring that presence of a quiet and dark room, using ear plugs and supplements such as melatonin, valerian root, lavender, magnesium and L-theanine.^14^

## Positive Family and Social Connections

The principle *‘Keep strong family ties’* proposed by Poulain and Herm highlights the important role of family integrity and support of the elders in the BZ communities.^1^ The foundation of “Family” can be interpreted in many ways depending on the country of residence, beliefs, marital status and many more. BZs have established family as a highly valued institution which their lives depend upon, is a way of coping, having fun and a mode of commitment. The value of family was studied by Hitchcott et al in Sardinia and highlighted the correlation of family and superior mental health which can impact several aspects of psychosocial factors. The study also intercalates how longevity can be also attributed to the value of family which provides novel effects on mental health from close interpersonal relationships.^61^

In the Ikarian study by Panagiotakos et al^25^ the majority of the study’s population was living together with someone else, usually spouse or a relative minimizing the feelings of loneliness.^25^ Consistently, Poulain et al^3^ showed that Sardinian elders were statistically significantly less commonly widowed (*P* = .003) and more commonly currently married (*P* = .034) compared to the rest of Italy.^3^

The ‘Moais’ as referred to by Okinawans is a social circle and behaviors that are shared between a small group of friends which supports a healthy mental state throughout life. Cornwell et al established the importance of socialization and the detrimental effects of social isolation. The lack of social connections is not always linked with feeling of loneliness or depression; however, both objective and subjective isolation worsen the physical and mental health of individual who were disconnected from social activities. The study by Cornwell et al reported that although older individuals may have subjective sense of isolation due to lowering their standards of social engagement, they have the same effects as individuals feeling the sense of loneliness.^62^ Smith et al also showed that loneliness in elders may contribute to the deterioration of functionality, poor quality of life and even higher mortality rates.^63^ The residents of the BZ are constantly participating in social activities and interactions. In a study for example by Suzuki et al it was shown that Okinawans were active in helping others.^64^ The frequent participation of the elders of BZ in community festivals and maintenance of a solid place in their villages’ communities indicates that the sense of support goes beyond the family. For the above reasons Poulain and Herm proposed the *principle ‘Stimulate strong community support’* as a factor of longevity in the BZ.^1^

### Recent Studies on the Association Between Social Engagement and Wellbeing and Longevity

A study by Zunzunegui et al provided evidence that family as a social engagement plays a crucial role as a predictor of survival for elder parents, which is associated with social recognition by children and enhancement of usefulness, mutual dependence and belonging in the social circle.^65^ Theeke et al reported a correlation between non-married status and a smaller number of people living in the household with loneliness. The above population also reported less exercise, more alcohol and tobacco use, greater number of chronic illnesses, higher depression scores and higher number of nursing home stays.^66^ Furthermore, a number of other studies have shown that marriage is associated with better health and protection against premature mortality, especially for males. The above has been supported by research on mortality variation by living arrangement among older adults.^3,67,68^

Yang et al studied the biological mechanisms through which social relationships impact health across the human lifespan. The findings of the study highlighted that disruptions and deficit in social relations could directly impact the progression of chronic diseases and disease onset as well as affect disease burden in late life. Therefore, they concluded that social relations provide a strong scientific basis for the prevention and intervention of disease and in promoting longevity.^69^ In a 9 year follow up study Berkman et al highlighted that both women and men that lacked social and community connections were 2 to 3 times more likely to die compared to other individuals that were more attentive in social events.^70^ The American Heart Association has also considered the above and has linked loneliness as a risk factor to CHD.^14^

### Positive Social Connections in BZ Compared to LM Recommendations

Based on studies from the BZ we can conclude that there is some evidence that positive family and social connections are particularly important, especially for elders, and may contribute to their overall health and mental status and even be a protective factor against CVD. New studies in the field of LM highlight the importance of maintaining social connections as part of the general wellbeing of the individual and these conclusions are consistent with the sociality of the inhabitants of BZ.^14^ However, future studies are needed in order to verify a possible association between positive family and social interactions and longevity, excluding possible confounders.

## Spirituality and Personal Motivation?

With the rise of the 21st century of technology and modern medicine, an increase in disbelief to religiosity has risen. For residents of BZ faith is an important aspect of their identity and daily life. Many recent studies propose that higher levels of individual religiosity can be associated with increased levels of personal satisfaction and happiness. All BZ have been linked with higher levels of community religiosity. According to Eichhorn et al, happiness drawn from the engagement in the religious beliefs of the community is partly due to the opportunities of social interactions and the positive feelings of shared beliefs in this context.^71^

Another study by Gebauer et al^72^ highlighted the cultural aspect of believing in a higher power was linked with higher individual self-esteem and psychological adjustment.^72^ Jackson et al also confirmed that religiousness and spirituality has a high impact on positive well-being outcomes and that spirituality has a greater and multidimensional aspect in comparison to religion itself.^73^ A large-scale cohort concluded that religiosity has been associated with decreased frequency of smoking and alcohol overconsumption, increased frequency of exercising and a decreased risk of diabetes mellitus and CVD.^74^ A study by Legrand et al in 71 Ikarians aged ≥90 showed that 90% believed in God and 81.4% participated in religious events.^9^ All of the above studies prove the positive correlation of “*Belonging”* as a motivational and emotional support system for all BZ residents.

The sense of ‘purpose’ has shown to be highly correlated with the long-lived individuals of BZ and thus the proposed principle ‘*Having a purpose in life’* as proposed by Poulain and Herm.^1^ Ikigai or “life worth living” is 1 of the most important psychosocial components of Okinawan’s living. ‘Ikigai’ is a subjective indicator of wellbeing, consciousness and joy. In a cohort study conducted by Sone et al^75^ they found that Japanese residents that had a lack of ‘ikigai’ were associated with a higher risk of mortality (mainly due to CVD), increased levels of CRP and pro-inflammatory cytokines and decreased levels of high-density cholesterol (HDL).^75^

### Studies Investigating the Association Between Motivation And/Or Spirituality and Longevity in the General Population

Based on some observations from the BZ, studies have been contacted to investigate the association between motivation and spirituality and longevity in the general population. In a study by Hill et al the researchers used data from the longitudinal Midlife in the United States (MIDUS) sample to examine whether purpose in life promotes longevity across the adult years. The results of the study established that purposeful individuals lived longer than non-purposeful individuals during a 14-year follow-up period, after elimination of confounders related to psychological and affective wellbeing. This highlights that the lack of purpose is an indicator of increased mortality.^76^

In another study by Kim et al assessed whether higher purpose in life among adequately functioning older adults was associated with lower risk of developing weak grip strength and slow walking speed over time. The study provided evidence individuals without a purpose developed a weak grip strength and slow walking speed. This finding supports that motivation and purpose in older individuals is positively associated with the maintenance of physical function.^77^ Finally, Tomioka et al conducted a prospective observational study which established that the lack in acquiring hobbies and a purpose in life (in Japanese Ikigai) was not only associated with increased mortality but also a decline in activities of daily living (ADL). The study also stressed out that hobbies and purpose increase cognitive function and reduce depressive symptoms.^78^

It should be noted that even though the pillar of spirituality and motivation is not specifically addressed by LM, currently there is limited evidence in the literature in this field and further research need to be conducted to examine further the association between *motivation and/or spirituality* and longevity.

## Respect for the Planet

Based on Poulain and Herm the habitants of each BZ show ecological concern and respect for the planet and this might even contribute to their wellbeing and longevity. Some of the possible explanations could be the reduced indoor and outdoor pollution, the presence of a balanced food chain and ecosystem and a healthy flora and fauna.^1^ It should be noted that the pilar *‘Respect for the planet’* is not addressed by LM. Currently, there is limited evidence in the literature and further studies should be conducted to examine the association between ecological concern and longevity.

## The Role of Genetics

Longevity is multifactorial and results from a combination of genetic and environmental factors. It has been noted that a healthy lifestyle may help individuals survive until their early 90s without major chronic diseases. However, genetics plays a major role in the transition to the supercentenarian age.^1^

Despite extensive research mostly 2 genes that have been strongly associated with longevity: FOXO3A and Apolipoprotein E2 (ApoE2) genotype.^79^ The ApoE2 genotype is known to be protective against Alzheimer’s disease and has also been associated with longevity. The study conducted by Poulain et al showed that this specific genotype is statistically significantly more prevalent in Ikarians in comparison to the rest of Greeks for both males and females. The same correlation was noted in Sardinian females. This study failed to show any statistically significant differences in the prevalence of other ageing related genotypes, such as the ApoE4 genotype, FOXO3A rs2802292 (TT genotype) or PON1 rs662 (RR genotype) between Sardinians and other Italians or Ikarians and other Greeks.^3^ Other prevalence studies amongst Okinawans have shown that the ApoE4 genotype, which increases the risk for Alzheimer’s disease, has a higher frequency of .005 amongst supercentenarians of Okinawa compared to the Japanese control of .097.^80^

There has also been a correlation between specific Human Leukocyte Antigen (HLA) haplotypes and longevity. Some examples include HLA-DQB1 and DQA1 in Okinawans and Sardinians and HLA-DRB1 in Sardinian centenarians; however further research is needed to derive specific conclusions.^81,82^ A surprising finding has been an association between genetic variants of the bitter taste receptor TAS2R38 and longevity in Sardinian populations.^83^ Even though many specific genes related to longevity are yet to be identified, there has been a strong association between family history and supercentenarian age. For instance, centenarians siblings in Okinawa have been shown to possess an increased probability of longevity and specifically a 2.58-fold increase for female siblings and a 5.43-fold increase for male siblings of reaching the age of 90 years.^84^ Poulain et al interestingly showed that among Sardinians the familial factor of exceptional longevity is strongly associated with the mother, especially for females, as well as the siblings, especially for males.^11^

One of the most crucial factors affecting the quality of life in the elderly population is mental function. There is substantial evidence highlighting the role of genetics in the development of Alzheimer’s disease and other types of dementia. Georgiopoulos et al studied the effect of Tumor Necrosis Factor alpha (TNF-α) and Angiotensin Converting Enzyme (ACE) polymorphisms in cognitive function in 178 residents of Ikaria which were 75 years of age or older. In the study, the researchers concluded that the DD genotype of ACE was linked to Alzheimer’s disease. Furthermore, individuals with a DD genotype for ACE and a GG genotype for TNF-α had an almost four-times increased risk of having a mini mental state examination score ≤24, which classifies the patient with dementia, after adjusting for various confounders. Additionally, aortic artery distensibility, an index of arterial aging and atherosclerosis were independently associated with the presence of the combination of the above genotypes.^85^

Finally, there is a need for more specific genome wide association studies to derive conclusive results regarding the association of specific genes to longevity.^3^

## The Effect of Behavior and Genetics on Body Systems and Molecular Pathways

Aging is a multifactorial process with affects practically all of the body systems and molecular pathways. The studies conducted have supported that lifestyle practices and genetics may protect the cardiovascular, immune, gastrointestinal, and endocrine systems of inhabitants in the BZ compared to those of the general population.

### Cardiovascular System

Pulse wave velocity is a marker of aortic stiffness which increases with old age and appears to be an independent predictor of all-cause and cardiovascular mortality. Pietri et al showed that Ikarians aged over 50 years old showed a decelerated decrease of pulse wave velocity in relation to the general population’s standards, which may contribute to the populations’ longevity.^86^

Pulse pressure is defined as the difference between systolic and diastolic blood pressure and it is elevated in individuals with aortic stiffness. Heart rate variability is defined as the variation of heart rate with breathing and it decreases with old age. Chrysohoou et al showed that Ikarian elders with a decreased heart rate variability had elevated values of pulse pressure, which may indicate that autonomic nervous system dysfunction may be associated with aortic stiffness and thus all-cause and CVD mortality.^87^

Intima-media thickness is a ultrasonographic measure of the thickness of an atherosclerotic plaque. Tatsukawa et al concluded that middle aged Okinawans had a statistically significantly lower intima-media thickness in comparison to the middle-aged residents of a suburban area of Fukuoka area in Kyhshu.^80,88^

Interestingly, arterial hypertension is highly prevalent amongst centenarians inhabiting Okinawa, Ikaria and Nicoya. As controversary as it may seem, there are indications that arterial hypertension might be a biological marker of better health and functionality in centenarians. Arterial hypertension has been associated with better physical and cognitive functions in Okinawans. Furthermore, in the scenario of a stroke or acute coronary syndrome, a hypotensive individual is more prone to ischemic damage and a slightly elevated blood pressure could possibly increase the survival in the centenarians with a major cardiovascular event. Madrigal-Leer et al stated that arterial hypertension was highly prevalent amongst Nicoyan elders. Legrand et al noted that 70.4% of the Ikarians from the study were hypertensive. However, the fact that the majority of these hypertensive Ikarians are well controlled may indicate the importance of the management of arterial hypertension in the risk of developing CVD.^7,49,80,89^

Coronary artery calcium is a measure of subclinical atherosclerosis but it has also been associated with the risk of malignancy and all-cause mortality. Lakshmanan et al showed that the prevalence of zero coronary artery calcium was statistically significantly (*P* <.001) more common in the Beach Cities of California than the rest of the state, which could constitute a BZ candidate.^90^

### Immune System

Serum neopterin is an immunological marker related to the activation of monocytes/macrophages which may be elevated in a variety of conditions, including viral infections, autoimmune diseases, graft rejection and malignancies. Besides, increased levels have also been associated with older age and it can serve as a predictive marker of all-cause mortality. Sotgia et al^91^ highlighted that neopterin levels in Sardinian centenarians was comparable to those aged 80-90 and significantly lower than those aged 95 years old, which may indicate an implication of the monocyte/macrophage system with longevity.^91^

### Gastrointestinal System

The intestinal flora has gained a great amount of attention in the past decade and has been correlated with various physiologic and pathologic states. One of the effects of ageing is the appearance of quantitative and qualitative changes in the intestinal flora. A study conducted by Biagi et al in Sardinians showed that bacteria from *the Ruminococcaceae*, *Lachnospiraceae*, and *Bacteroidaceae* families, which usually decrease along with age, persisted in great numbers in centenarians aged 105-109 years old.^92^ Wu et al also highlighted that centenarians have a unique pattern of bacteria in their intestine: decreased numbers of *Faecalibacterium prausnitzii* and *Eubacterium rectale* and increased numbers of *Methanobrevibacter smithii* and *Bifidobacterium adolescentis*. The later study also stated that these bacteria possess a metabolically active role in the intestine of supercentenarians characterized by glycolysis and fermentation to short-chain fatty acids, which is considered beneficial for the host.^93^

### Endocrine System

Centenarians inhabiting Okinawa have been reported to have a decreased prevalence of diabetes mellitus and a favorable lipid profile, especially HDL. The decreased prevalence of diabetes mellitus in elders of the BZ was confirmed by the study of Madrigal-Leer et al in Nicoyans.^49^

It is well known that the prevalence of metabolic syndrome increases in older ages. One of the possible causes in men is the gradual decline in the levels and function of free and total testosterone with ageing.^94^ This may lead to insulin resistance, central obesity and increases in body fat, lipid profiles and inflammation.^95^ There has been a study conducted in elders of Ikaria (n = 467, 75 ± 6 years old, 47% men) which concluded that male participants with a testosterone level >407 ng/dL were younger and had a lower prevalence of metabolic syndrome than those with testosterone levels <289 ng/dL.^94^ The relationship associating low testosterone levels to a predisposal for metabolic syndrome was statistically significant only in men. Specifically, a 100 ng/dL increase in total testosterone was associated with a 10% decreased risk of metabolic syndrome, after excluding other possible confounders. Besides total testosterone, the age-dependent increase in Steroid Hormone Binding Globulin levels by 1.2% per year leads to a decline in free testosterone and may also play a major role in the increased risk of metabolic syndrome in elder males.^95^ Besides metabolic syndrome, testosterone deficit has been associated with the development of type 2 diabetes mellitus, sexual dysfunction, anemia, mental disorders (eg, major depression), fatigue, sarcopenia, osteoporosis and various other manifestations in elderly men.^96-98^

A study conducted by Tolu et al concluded that there is a statistically significant relationship between the presence of endemic goiter and longevity in Sardinians and this has been verified by animal studies and cross-sectional studies in other BZ. However, the exact mechanism or the significance of this finding remains inconclusive.^99^

### Molecular Pathways

From a molecular aspect, ageing is a result of chronic cellular hyperfunction, which leads to the accumulation of damage to the vital macromolecules (eg, DNA, proteins, lipids) disrupting their normal function and leading to age related diseases (eg, CVD, cancer). Research has correlated many molecular pathways/processes and key players to be associated with cellular hyperfunction/dysfunction and ageing and these include chronic inflammation, reactive oxygen species (ROS), telomere length, DNA methylation, the IGF-1 and mTOR pathways, AMPK, NO and various miRNAs.^100^

Reactive oxygen species can cause direct damage to vital cellular macromolecules and lead to cell dysfunction and age-related diseases. Suzuki et al concluded that the plasma levels of lipid peroxide of Okinawan centenarians, an index of oxidative stress, was significantly lower compared to younger controls. This could be indicative that longevity may be associated with protective mechanisms against oxidative stress in centenarians.^100^

Various studies have linked declines in telomere length with the process of ageing. Rehkopf et al^101^ studied the leukocyte telomere length in Nicoyans and concluded that these individuals had a statistically significantly longer telomeres (81 base pairs) in comparison to inhabitants of other areas of Costa Rica, which could contribute to the longevity of the former.^101^ Furthermore, the Costa Rican Longevity and Healthy Aging Study surprisingly showed that specific dietary patterns, such as rice and beans, were associated with longer leukocyte telomeres.^102^ Finally, DNA methylation patterns, which have also been associated with ageing, have been shown to differ between Nicoyans and non-Nicoyan residents of Costa Rica, indicating a possible correlation between specific patterns and longevity.^103^

These pathways are affected by a variety of behavioral and genetic factors as already analyzed in the paper, for instance caloric restriction affects the mTOR and IGF-1 pathways, vegetarian diets may affect a variety of miRNAs and telomere length, red wine possess anti-oxidant effects and exercise regulate endothelial function and NO secretion. One strategy to decelerate the process of ageing could involve the targeting of specific molecular pathways to prevent the development of cellular hyperfunction and dysfunction. Some promising interventions include mTOR inhibitors (eg, everolimus, sirolimus, caloric restriction), metformin (glycemic control, AMPK activation), statins (anti-lipidemic drugs with pleotropic functions), aspirin (anti-inflammatory and anti-platelet effects) and medications with anti-hypertensive and cardioprotective effects (lisinopril, propranolol and tadalafil).^104^ Inhibition of mTOR via medications or dietary modifications (ie, caloric restriction) constitutes a promising anti-ageing intervention. However, in regards to mTOR inhibitors, further research is required, including randomised controlled trials, in order to investigate the ideal dosage, confirm the efficacy and evaluate the safety profile for use in the prevention of age-related diseases and expansion of lifespan.^16,104-106^

## Conclusions

**Figure 1.**
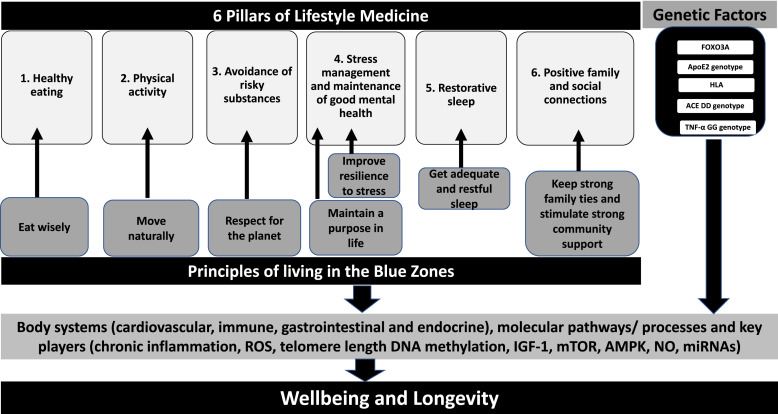
Lessons learned from the Blue Zones, the 6 Lifestyle Medicine Pillars and recent results from the literature support the association between healthy eating, physical activity, avoidance of risky substances, stress management and maintenance of good mental health, restorative sleep and positive family and social connections and wellbeing and longevity. Future studies should assess the combined effects of behavioral and genetic factors on molecular pathways to understand their contribution to longevity.

The BZ are great examples of communities with characteristics favoring a prolonged life expectancy. By comparing the lessons learned from the BZ (*move naturally, eat wisely, avoid stress, get adequate and restorative sleep, keep strong family ties, stimulate strong community support, respect for the planet* and *having a purpose in life*), to the 6 Pillars of LM and by reviewing recent research it is evident that *healthy eating*, *physical activity*, *avoidance of risky substances*, *stress management and maintenance of good mental health*, *restorative sleep* and *positive family and social connections* are associated with wellbeing and longevity (Figure 1).

In addition to the role of behavior, genetics seems to play an important role in affecting longevity and some of the genes involved include FOXO3A and ApoE2 and HLA. Both behavioral and genetic factors influence the cardiovascular, immune, gastrointestinal and endocrine systems and further research should be conducted to investigate the molecular pathways and key players involved. Current research supports a role for chronic inflammation, ROS, telomere length, DNA methylation, IGF1 and mTOR pathways, AMPK, NO and miRNAs in affecting longevity (Figure 1).

Despite the preliminary evidence derived from observational studies in the BZ, up to date there has not been a specific scientific explanation regarding the exceptional advantage of survival of the inhabitants of longevity BZ which is likely the result of a combination of various behavioral and genetic factors.^2,3,5-11^ Therefore, there is an urgent need for conducting in depth interdisciplinary comparative studies in the BZ and coupled with the implementation of comprehensive lifestyle behavior intervention studies in the general population. Such studies should attempt to also investigate which behavioral and genetic factors and which molecular pathways are key players associated with wellbeing and longevity. The results of such studies will help us understand both the contribution but also the interaction between behavioral and genetic factors but will also shed further light to the mystery of the BZ and further enhance the LM recommendations for healthy living.
